# Antimicrobial Stewardship: A Review for Internal Medicine Physicians

**DOI:** 10.7759/cureus.14385

**Published:** 2021-04-09

**Authors:** Syeda Sahra, Abdullah Jahangir, Vincent De Chavez

**Affiliations:** 1 Internal Medicine, Staten Island University Hospital Northwell, Staten Island, USA; 2 Internal Medicine, Staten Island University Hospital, Staten Island, USA; 3 Infectious Diseases, Staten Island University Hospital, Staten Island, USA

**Keywords:** antimicrobial stewardship program, anitibiotics, super bugs, antimicrobial resistance

## Abstract

Antimicrobial stewardship is the need of the hour to prevent the collapse of our health care system at the hands of a pandemic of resistant pathogens. Inappropriate and indiscriminate abuse of antibiotics has left very few options for prescribing physicians as most of the pathogens, particularly gram-negative, are resistant to the major antibiotics. This article reviews the importance of Antimicrobial Stewardship Programs (ASP) for internal medicine physicians and residents. Commonly encountered clinical scenarios are discussed. Appropriate indications of antibiotics, pathogen-guided prescriptions, adverse effects of common antibiotics, and options to use newer antibiotics are reviewed. The role of a health care team is highlighted. The evidence-based steps taken to ensure ASPs implementation are reiterated to serve as an educational guide for medical residents and physicians.

## Introduction and background

Antimicrobial resistance and need for antimicrobial stewardship

Antibiotics have achieved historical success against microbes since the discovery of penicillin. Soft-tissue infections and abscesses were previously thought deadly and wiped out populations during World War 1 and 2. Their repeated use and improvement of infections built the trust of clinicians and patients alike. The advancement in molecular biology and decoding of the bacterial genome in the last century introduced many ideas leading to sophisticated antibiotic production and use. Nevertheless, the indiscriminate and injudicious use of antimicrobials has contributed to antimicrobial resistance.

Newer strains of pathogens, particularly gram-negative bacteria, have developed advanced resistance mechanisms to traditional antibiotics. The pathogenic targets of antibiotics have evolved within a short time and over a few successive generations. Bacteria have improvised newer mechanisms to counter the antibiotics available in our health care systems. Modification of penicillin-binding proteins, efflux pumps, and production of beta-lactamases has rendered a lot of antimicrobials inefficient. Carbapenems were introduced due to their structural ability to have some protection against beta-lactamases. They became the empiric therapy of choice for patients presenting with sepsis or serious infections, and now carbapenem resistance is also on the rise. Pathogens adapted to the changing environment and antibiotics by several mechanisms (Table 1).

**Table 1:** : Common mechanisms adopted by pathogens to resist antibiotics

Common mechanisms adopted by pathogens to resist antibiotics
Enzyme mediation degradation of active pharmacological agent (e.g., carbapenemases, New Delhi Metalloproteases, OXA-48 )
Changes in cell permeability to block or decrease the entry of antibiotics
Efflux pumps
Modification in receptor sites to influence the activation/inhibition of receptor targets
Intrinsic resistance mechanism including but not limited to upregulation of metabolic pathways favoring resistance

*Enterococcus faecium*, *Stenotrophomonas maltophilia*, *Staphylococcus aureus*, carbapenem-resistant *Acinetobacter baumannii*, *Pseudomonas aeruginosa*, carbapenem-resistant enterobacteriaceae (CRE), and extended-spectrum beta-lactamases (ESBL) are particularly incriminated in recurrent infections and nosocomial breakouts in health care centers. ESKAPE pathogens (*Enterococcus faecium*, *Staphylococcus aureus*, *Klebsiella pneumoniae*, *Acinetobacter baumannii*, *Pseudomonas aeruginosa*, and *Enterobacter* species) have become havoc for health care systems. These resistant organisms, also termed 'super bugs', contribute to prolonged hospitalization, inpatient morbidity, and mortality and impose a heavy burden on health care systems universally. These pathogens now warrant potent agents to counter their resistance mechanisms. Despite the research and development efforts in the recent past by pharmaceutical companies, no new significant antibiotic generation has made it to the market. Mindful use of the antibiotic options available can help prevent the burdened health care system from collapsing by the pandemic of resistant and recurrent infections. Antimicrobial stewardship might be the only way to fight against infectious diseases until more potent antibiotics are available.

The term 'antimicrobial stewardship' was first used in the late-1990s. It refers to a set of coordinated efforts for appropriate antibiotic selection, the correct dosing of selected antibiotics, and de-escalation to a more straightforward and oral regimen when clinically applicable with the shortest yet effective length of treatment. When implemented, the outcome of Antimicrobial Stewardship Programs (ASPs) is minimal drug toxicity and adverse events, best clinical results, and avoidance of antibiotic resistance. Pathogen-directed antibiotic therapy is used to treat the infection and prevent recurrence effectively. The organized mediation to identify pathogen susceptibility and use of targeted antibiotics to prevent resistance is the foundation of ASPs [1-3].

The clinicians should know when to use an antibiotic and when not to. They should be able to select the most appropriate antibiotic based on the pathogen. The pharmacists, microbiological lab, and infection control simultaneously guide and support the decision-making for selecting antibiotics, dosage, and administration routes to get the most optimum clinical results. These efforts prevent the recurrence of the infection by the same bug and resistance against the antibiotic in question.

The proper implementation of ASP protocols can prevent the growing pathogen resistance and preserve existing antibiotics' efficacy [4]. 

One Health is a global collaboration based on interdisciplinary efforts to optimize infection control measures and antibiotics use in humans and animals. The end goal is better health outcomes for humans, animals, and the environment [5]. 

## Review

Measures taken to establish antimicrobial stewardship

Initiation of appropriate broad-spectrum antibiotics can be the difference between life and death for patients presenting to the Emergency Room. Regional susceptibility and the patient's risk factors should be kept in mind. Similar clinical presentation in a young patient with no prior medical history and another elderly patient with uncontrolled co-morbidities can warrant different antibiotics to cover different pathogens.

Risk assessment of patients

Risk factors in a patient for MRSA (Methicillin-Resistant *Staphylococcus aureus*) and *Pseudomonas aeruginosa *should be identified and taken into account while selecting antibiotics. Both MRSA and *Pseudomonas *infections predispose patients to an increased hospital stay, morbidity, and mortality. The isolates of *Pseudomonas *are found to have intrinsic resistance against many antibiotics. The isolates are further classified into multi-drug resistant, extensively drug-resistant, and pan-sensitive groups based on their increasing resistance to the majority of antibiotics available. Risk factors for MRSA include prolonged hospitalization and stay in critical care units and nursing homes, prior colonization of MRSA, invasive procedures, immunosuppressive health states, hemodialysis, and presence of chronic central venous catheters. Similarly, patients with diabetes mellitus, bedridden functional status, stay in surgical and critical care units, invasive catheters or devices, and exposure to prior broad-spectrum antibiotics (cephalosporins, carbapenems, fluoroquinolones, aminoglycosides) are at a high risk of *Pseudomonas *infection.

Mechanism of action of drugs should be kept in mind. Daptomycin has excellent MRSA coverage, but it is not used in pulmonary infections due to its inhibition with the surfactant, rendering it ineffective against respiratory pathogens.

The criteria-based authorization and approval of essential drugs by the Infectious Diseases team has proved successful. The consultant should assess the clinical need. Infectious diseases consultants should determine rational versus compassionate use on a case-to-case basis. We saw an example of this rationing in the earliest era of the COVID-19 pandemic, where hydroxychloroquine, tocilizumab, and convalescent plasma were used before FDA (United States Food and Drug Administration) approval. It led to a shortage of these medications and exposed a considerable population to adverse events as well. Hydroxychloroquine is known to cause QTc interval and can potentially lead to cardiac arrhythmias. The use of tocilizumab leads to an increased predisposition to fulminant fungal infections.

Similarly, the use of convalescent plasma in patients at the brink of volume overload due to underlying cardiac or renal nephropathy can be drastic. Thus, an appropriate and responsible use of these medications based on the phase and severity of illness determined by the Infectious Diseases and Medicine teams can significantly improve clinical outcomes.

Role of the Infectious Diseases team in the selection and dosing of appropriate antibiotics

The Infectious Disease team should be on board for appropriate antibiotic selection and recommending the correct dose and length of treatment to secure the targets of minimal toxicity and best clinical outcomes.

Intravenous drugs should be changed to oral when appropriate as it decreases the chances of catheter-associated infections. Health care systems are flagged by the health departments based on catheter-associated infection reports and suffer from losing reimbursement. The patients always prefer the oral route, which shortens the hospital stay and drug costs. Particularly, during this current era of an ongoing pandemic, efforts should be made to avoid an unwarranted hospital stay. Agents with equivalent oral bioavailability like linezolid, bactrim, fluconazole, moxifloxacin, and clindamycin should be used orally when applicable. These antibiotics can ensure similar serum concentrations and efficacies.

Appropriate institutional protocols for initiating empiric antibiotics should be in place based on regional susceptibility and resistance patterns. The Infectious Diseases team should follow evidence-based guidelines for the duration of therapy. The treatment duration in gram-positive and gram-negative bacteremia is also defined based on the pathogen encountered to prevent unnecessarily prolonged treatment duration. Newer studies have indicated the traditional treatment course for osteomyelitis for six weeks can be replaced with four weeks. Similarly, oral agents with good bone penetration are also being introduced, potentially avoiding long-term catheters [6].

Infectious Diseases consultation should always be on board whenever resistant pathogens like *Enterococcus faecium*, *S. aureus*, *Klebsiella pneumoniae*, *Acinetobacter *species, *Pseudomonas aeruginosa*, *Enterobacter* are encountered for appropriate pathogen-directed therapy with minimal adverse events, resistance, and optimal clinical outcomes.

The importance of recognizing pathogen-targeted therapy

A single antibiotic is preferable to two antibiotics when similar coverage is desired. The use of two different antibiotics for covering the same spectrum, e.g., gram-negative rods or anaerobes by both beta-lactams and fluoroquinolones, should be discouraged. Double coverage leads to unnecessary antibiotic exposure and its adverse effects. Similarly, physicians can select a combination of antibiotics if the synergistic effect is clinically potent and shortens the action duration. In a healthy individual with community-acquired pneumonia, a macrolide alone is sufficient. Cephalosporins and macrolides are both initiated in patients with clinical severity to cover all possible pathogens. The ultimate goal of initiation of broad-spectrum coverage is immediate de-escalation as soon as possible based on microbiological results and clinical response.

De-escalation and simplification of antibiotic regimen

De-escalation of antibiotics to simpler regimens with a shorter yet effective course is performed based on results from cultures, antibiograms, and patient's response (back-end approach). Vancomycin, for example, is used to cover the gram-positive *Staphylococcus *empirically. If the *Staphylococcus *is not MRSA, clinicians should de-escalate the antibiotics to nafcillin or cefazolin to target methicillin-sensitive *Staphylococcus aureus*. This strategy prevents the unnecessary use of vancomycin and avoids its adverse effects. 'Front-end approach' (limited availability of antimicrobials most commonly associated with resistance) should be employed. The Infectious Diseases team should start a narrower pathogen-directed regimen promptly once susceptibilities are determined [7].

Monitoring response and ensuring compliance

The floor team should monitor the clinical response with vital signs and relevant blood work for hospitalized patients. Outpatient clinics should acquire blood cultures and markers of inflammation to guide the treatment course where appropriate. For example, daptomycin is known to cause muscle injury and rhabdomyolysis. Clinicians should monitor creatine phosphokinase (CPK) levels every week. The drug should be discontinued if a patient develops significantly high CPK levels or muscle weakness and pain with moderate CPK elevation.

Serum levels are measured and monitored for certain antibiotics (vancomycin) based on the patient's weight and renal function. Drug doses are adjusted accordingly to maintain adequate concentration and avoid adverse effects.

Directly observed therapy (DOT) is employed for tuberculosis patients to ensure antituberculous medication compliance. DOT was traditionally performed in-person, but now compliance can be monitored remotely. The emergence of multi-drug resistance strains is prevented by ensuring the appropriate antituberculous treatment for the entire duration. 

Rapid testing of health care workers exposed to body fluids from patients with high suspicion of viral infections (HIV, hepatitis B, hepatitis C) is ensured in health care settings. Initiation of timely antivirals prevents long-term complications, including immunosuppression and liver cirrhosis.

The timely availability of HIV antiviral therapy for pre-exposure and post-exposure to high-risk populations (sex workers, men who have sex with men) is also essential to decrease the viral load and prevalence of HIV in the community.

The threat of *Clostridiodes difficile*

The most significant risk factor for developing *C. difficile *infection is antibiotic exposure. Other risk factors include advanced age, recurrent hospital stay, residence in long-term health care facilities, chronic co-morbidities, and immunosuppression.

Antibiotics notorious for *C. difficile *should be recognized (cephalosporins, clindamycin ampicillin, fluoroquinolones, amoxicillin). Their use should be minimized, and patients should be monitored for gastrointestinal symptoms.

The concept of antimicrobial stewardship becomes especially important when we consider that *C. difficile* is responsible for the number one cause of nosocomial diarrhea and consequent morbidity and mortality. If appropriate antibiotics are prescribed per indication without prolonging the length of treatment, the health care burden can be easily decreased. Hand hygiene with isolation and contact precautions are the cornerstone in the prevention of *C. difficile *infections; this is where the collaboration with nursing reports and infection control measures becomes paramount. The role of internal medicine physicians and trainees should be to be mindful of unwarranted antibiotic exposure. They should be able to identify the threat of C*. difficile *and effectively treat and prevent its potential outbreaks by following the general principles of antimicrobial stewardship. The antibiotics reported with increased prevalence of *C. difficile *infection and disease should be recognized at institutional levels and their use should be restricted. 

If diarrhea persists in the absence of any laxative use, the suspicion for *C. difficile *should be very high. Stool polymerase chain reaction (PCR) testing for *C. difficile *should be carried out, and the patient should be placed on contact isolation until negative testing is obtained. If positive, the patient should be treated with oral vancomycin. *Clostridiodes difficile *can complicate into megacolon with the risk of possible colon rupture and sepsis. Intravenous metronidazole is used in fulminant infection. Surgery should be on board if there is a suspicion of ileus or toxic megacolon [8].

Proper indication to use antibiotics

Unwarranted use of antibiotics contributes to resistance at all levels. Studies have shown that a significant proportion of antibiotics prescribed are without proper indication. The clinical conditions most encountered by internists and primary care providers need to be probed as most need supportive care and not the potentially broad-spectrum antibiotics.

The emergency team should assess the patient presenting to the Emergency Room for sepsis per the sepsis criteria based on Sequential [sepsis-related] Organ Failure Assessment (SOFA) scoring. Blood work, including cultures, should be drawn and followed. The Infectious Diseases team should de-escalate antimicrobial therapy as quickly as relevant clinical results became available. 

There are several common clinical scenarios encountered in everyday practice where the use of antibiotics becomes questionable.

Asymptomatic bacteriuria (except in pregnant females and patients undergoing genitourinary intervention) does not need antibiotic treatment. Clinicians should interpret urine analysis carefully. The microbiological laboratory should count in the number of bacteria and white blood cells and leukocytic esterase and nitrite. Clinicians should take the presence of a chronic foley catheter into account as it can lead to a positive urine analysis due to urinary stasis. Genitourinary symptoms (burning and pain while passing the urine) and physical examination of the abdomen should be the deciding factor for treating a positive urine analysis [9]. 

Similarly, soft tissue infections need to be appropriately identified to determine the element of infection. Clear delineation between cellulitis and chronic dermatitis based on clinical exposure can prevent antibiotic usage and subsequent resistance. Chronic skin changes and lower extremity lymphedema can often mimic cellulitis, which does not need antibiotic treatment. Physicians should distinguish between purulent and non-purulent cellulitis to determine the most appropriate antibiotic course.

Viral pneumonia should not be covered empirically with antibiotics and require only supportive measures in immunocompetent people. The initial clinical presentation of gout and superficial bursitis can be mistaken for septic arthritis with broad-spectrum antibiotic coverage. The serum uric acid levels are not diagnostic during an acute attack of gout, and the standard or lower uric acid levels cannot rule out gout. The clinicians should be well-versed in the clinical presentation and risk factors for gout (red meat and shellfish, alcohol, certain medications, family history, age, and gender) to make the correct diagnosis.

Adverse drug reactions

The adverse effects of antibiotics need to be kept in mind while prescribing them to patients in all settings. Apart from allergic reactions ranging from hives and rashes to anaphylaxis, there are risks of significant renal and hepatic injury with many antibiotics. Penicillins and cephalosporins are associated with allergic reactions. Certain macrolides and fluoroquinolones are known to cause prolongation of QTc interval (macrolides, fluoroquinolones, hydroxychloroquine) and can lead to life-threatening arrhythmias. These arrhythmias, including Torsades de pointes, can be fatal. QTc interval should be obtained at baseline and followed in high-risk patients. Clinicians should minimize the use of QTc interval prolonging antibiotics in patients with cardiovascular co-morbidities [10]. Clostridiodes difficile, mentioned previously, is an opportunistic pathogen that multiplies exponentially when the healthy gut microbiome is disturbed by using antibiotics. Immediate identification of *C. difficile *infection and treatment is paramount to prevent the burgeoning morbidity from nosocomial diarrhea [11]. Fluoroquinolones should be used cautiously in the elderly population because they tend to potentiate tendinopathy. Aminoglycosides are notorious for injury to kidneys and the eighth cranial nerve (loss of hearing and balance). Chloramphenicol is known to suppress the bone marrow. Tetracyclines adversely cause photosensitivity and rashes. They also damage enamel, especially in children. Vancomycin is known for renal injury and should be used cautiously. Dosage of such nephrotoxic drugs should be appropriated to their kidney function and approved by Clinical Pharmacists. Vancomycin also causes widespread erythema and pruritis called 'Redman Syndrome' and is essentially an infusion-related reaction. Hepatic toxicity is a known side effect of many antibiotics, including antituberculosis drugs.

Newer combination and glycopeptide antibiotics: a ray of hope

Newer antibiotics are being introduced to circumvent the growing problem of resistant pathogens. Combinations of antibiotics with different mechanisms of action are used to create a synergistic effect against pathogens. Pathogens developed mechanisms like regulation of efflux pump and porin proteins to influence antibiotics' permeability and entry. They also produce enzymes, including but not limited to beta-lactamases and carbapenemases, which counteract the antimicrobial efficacy. Combinations with newer enzyme inhibitors are being introduced to cover the spectrum of resistant pathogens. The use of newer antibiotics like ceftazidime-avibactam and ceftolazone-tazobactam should be encouraged to cover most resistant pathogens, including beta-lactamase ESBL carbapenem-resistant Enterobacter (CREs). Ceftaroline is being preferred for MRSA and *Streptococcus pneumoniae* coverage. It is a fifth-generation cephalosporin used for community-acquired bacterial pneumonia and soft tissue infections [12]. Dalbavancin is a newer lipoglycopeptide covering gram-positive cocci, including *Staphylococcus aureus*. It is administered intravenously once weekly, making it attractive and convenient for the patients [13]. Oritavancin is also used for gram-positive organisms. It covers MRSA and vancomycin-resistant *Enterococci *(VRE). This is available as a one-time dose and does not need frequent monitoring [14]. Colistin has good gram-negative coverage and is available in intravenous as well as aerosolized form [15].

The newer antibiotics are expensive. The insurance does not cover the newer antibiotics in most cases. They are also in limited supply and short stock in many parts of the world. Physicians should keep the individual adverse effects of these antibiotics as well. For example, colistin causes severe nephrotoxicity. Newer antibiotics have limited safety and efficacy data in specific patient populations, including pregnant and lactating females, children, and elderly patients. More research is warranted, but these newer drugs are promising and used wherever appropriate. The dependence on traditional antibiotics would decrease and prevent further antimicrobial resistance.

How can internal medicine physicians and health care team contribute to ASPs

The health care team ensuring implementation of ASPs comprises Infectious Diseases consultants, resident and fellow physicians, hospital pharmacists, microbiology laboratory, infection control department (hand hygiene, contact precautions, bundles of care), and clinical pharmacists.

Educational interventions to assess the physician's understanding of antimicrobial resistance patterns and adverse events have been tried in the past. These interventions include providing information regarding drug resistance and toxicity and reinforcing updated antimicrobial guidelines for future prescribing. Management of complicated infections in ICU settings based on evidence-based guidelines and algorithms to use optimum empiric coverage is discussed. Individual risk factors for MRSA infection should be recognized (antibiotics exposure, hospitalization) by physicians. Eligible patients should be swabbed for MRSA PCR testing. Nasal mupirocin should be used for decolonization. Decolonization interventions dramatically decrease the prevalence of MRSA infections [16, 17]. The purpose is to refresh the knowledge regarding current antimicrobial guidelines and infection control measures. Perioperative prophylactic antibiotics should be used appropriately and not prolonged unnecessarily. De-escalation of antibiotics to simpler regimens with a shorter yet effective course based on results from cultures, antibiograms, and patient's response (back-end approach) should be reiterated in medical rounds [18, 19].

Understanding and applying pharmacokinetics and pharmacokinetics to select appropriate antibiotics and adjust their dosing by the hospital and clinical pharmacists can avoid redundant antibiotics for double coverage. Accurate documentation and regular update of antibiotic allergies by nursing staff can help explore alternative antibiotics [20, 21]. 

Physicians, pharmacists, and nursing staff should confirm the documented allergy to penicillins before resorting to broad-spectrum antimicrobials. Most of the allergies are documented based on remote exposure with mild symptoms. Clarification of the allergic reaction and risk versus benefit analysis can help prevent the use of broad-spectrum alternatives. Aztreonam is used in cases of penicillin and cephalosporin allergies, but clinicians should limit its use to true allergies and cases where extended-spectrum coverage is desired [22].

The microbiological lab should carry out the organisms' drug susceptibility to avoid the use of resistant antibiotics. Diagnostic Stewardship leading to quicker identification of pathogens can help earlier implementation of targeted therapy. The antibiograms should have open access for clinicians and pharmacists. The data from antibiograms can be construed to predict future outbreaks or improve pathogen-load at the hospital level [23, 24].

## Conclusions

The 'Get Smart Campaign' was started in 1995 to limit the inappropriate antibiotic prescriptions in outpatient settings, followed by 'Get Smart in Healthcare' to achieve similar targets for hospitalized patients. The USA government has introduced a national action plan to counteract resistant pathogens (CARB, Combating Antibiotic-Resistant Bacteria) for 2020-2025, an extension of prior Antimicrobial Stewardship Programs based on federally governed interventions. The main goals are to ensure infection control measures and prevent antibiotic resistance by encouraging rapid pathogen detection and the development of newer antibiotics and vaccines. Centers for Disease Control and Prevention (CDC) has introduced the Antibiotic Resistance Solutions Initiative to strategize infections and resistance prevention. Efforts will be organized around prompt detection, reporting and containment of infection, improved antibiotic use, and newer antibiotics research. Surveillance bodies should be available to ensure the implementation of Antimicrobial Stewardship Programs. They assess the targeted health care systems, physicians' understanding and trends of antibiotics prescription, adverse drug reactions, cost-benefit analysis and purchase data of hospital pharmacies, and re-admission rates for recurrent infections before and after the implementation of ASPs. Protocols are changed depending on the feedback and clinical outcomes obtained. 
